# Crop bioengineering via gene editing: reshaping the future of agriculture

**DOI:** 10.1007/s00299-024-03183-1

**Published:** 2024-03-18

**Authors:** Mohamed Atia, Wenjun Jiang, Khalid Sedeek, Haroon Butt, Magdy Mahfouz

**Affiliations:** Laboratory for Genome Engineering and Synthetic Biology, Division of Biological Sciences, 4700 King Abdullah University of Science and Technology (KAUST), 23955-6900 Thuwal, Saudi Arabia

**Keywords:** CRISPR/Cas system, Crop engineering, Trait engineering, Food security, Genome editing

## Abstract

Genome-editing technologies have revolutionized research in plant biology, with major implications for agriculture and worldwide food security, particularly in the face of challenges such as climate change and increasing human populations. Among these technologies, clustered regularly interspaced short palindromic repeats [CRISPR]–CRISPR-associated protein [Cas] systems are now widely used for editing crop plant genomes. In this review, we provide an overview of CRISPR–Cas technology and its most significant applications for improving crop sustainability. We also review current and potential technological advances that will aid in the future breeding of crops to enhance food security worldwide. Finally, we discuss the obstacles and challenges that must be overcome to realize the maximum potential of genome-editing technologies for future crop and food production.

## Introduction

Providing food and nutritional security requires adequate production of cereal, vegetable, and oilseed crops. However, current agricultural practices struggle to keep up with rising demand (Foley et al. 2011; Ray et al. 2013). Moreover, extreme weather events caused by climate change, such as droughts, floods, heat waves, and shifting precipitation patterns, pose significant threats to crop yields and overall agricultural productivity. In addition, the limited availability of arable land and increases in soil degradation, water scarcity, pathogens, and parasitic infestations related to climate change exacerbate these issues.

The acceleration of crop improvement programs through biotechnology and breeding has led to the discovery of crucial traits for the production of improved varieties that could ameliorate the negative consequences of population increase and climate change. These target traits include increased yield and tolerance of biotic and abiotic stress, with drought, heat, and salt tolerance being particularly important, along with broad, durable resistance to diverse pathogens. Additional target traits focus on agricultural inputs (nitrogen use efficiency and usage of other nutrients, water use efficiency, herbicide tolerance), photosynthetic efficiency, plant architecture, and on so-called output traits, such as lipid composition. Indeed, modern biotechnology allows researchers to manipulate key enzymes in specific metabolic pathways, which can be used to increase the levels of desirable nutrients such as vitamins and iron while decreasing the levels of unfavorable compounds such as phytic acid and acrylamide-forming amino acids. Although many modifications have shown promise in the lab, improving agronomic traits requires communication between breeders and molecular biologists to identify interventions that avoid tolerance–yield tradeoffs and produce changes that improve crop production under actual field conditions (Merritt Khaipho-Burch 2023).

Crop breeding and molecular biology methods have become increasingly intertwined, as conventional breeding gave way to molecular breeding, which eventually evolved, after the Green Revolution era, into genomics-assisted breeding. Advances in high-throughput genomics procedures at the whole-genome level, such as genetic association mapping, map-based cloning, genomic selection, and speed breeding, have been effective in accelerating crop development. Similarly, genetic engineering tools such as gene cloning, overexpression, and knockout and knockdown have diversified into new techniques for increasing, or decreasing gene function, including RNA interference (RNAi) and virus-induced gene silencing (VIGS).

Here, we focus on gene and genome editing, which can be used to generate lines with improved agronomic and climate-resilient traits (Gao 2021). Specifically, we shed light on ongoing efforts to apply gene-editing techniques to crop improvement. We provide an overview of the strategies pursued thus far and discuss the constraints and prospects inherent in gene-editing technologies.

## Plant genome-editing technologies

A new era of genome engineering initiated by the development of genome-editing technologies is allowing the alteration and targeting of specific DNA sequences to modify plant genomes efficiently, accurately, and quickly (Jaganathan et al. 2018). Introducing mutations into a target DNA sequence usually involves three steps. The first step is the recognition of the target DNA sequence by an externally designed nuclease composed of a recognition module and a nuclease domain. In the second step, the nuclease binds to the target sequence and induces DNA double-strand breaks (DSBs) at or near the target location. The ability of the different genome-editing technologies to competently generate targeted DSBs enables a varied array of genetic outcomes (Osakabe and Osakabe 2015). In the third step, the DSB is repaired by endogenous nonhomologous end-joining (NHEJ) for error-prone genome repair or homology-directed repair (HDR) pathways for precise genome engineering (Altpeter et al. 2016). Several major genome-editing technologies have been developed and we describe these in the following sections.

### ZFNs and TALENs for plant genome editing

**Fig. 1:**
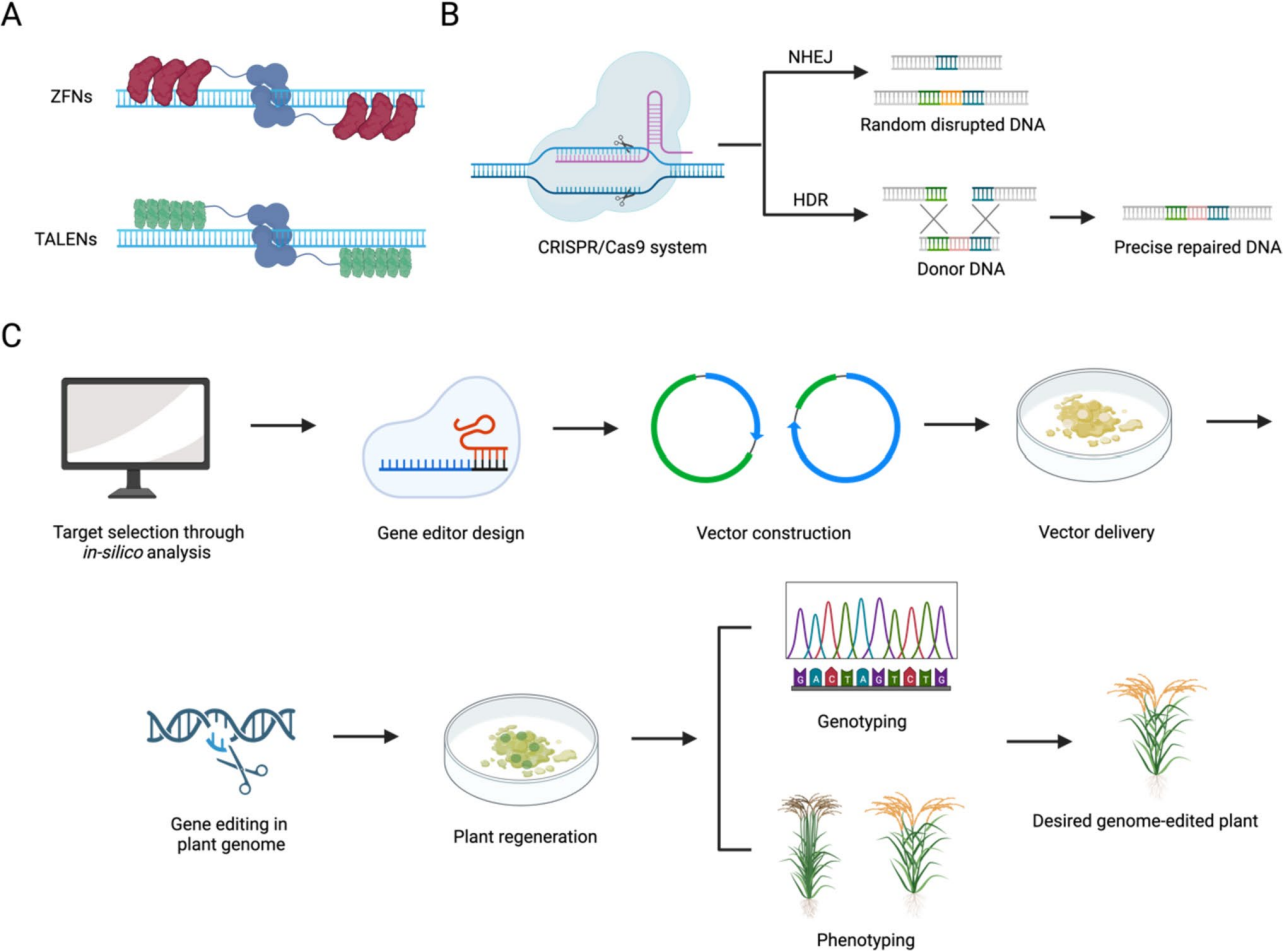
The tools and workflow of plant genome editing. **A** Zinc Finger Nucleases (ZFNs) and Transcription Activator-Like Effector Nucleases (TALENs) as plant genome editing tools. **B** CRISPR/Cas9 systems as genome editing tools; CRISPR/Cas9 mediated DSB can be repaired by error-prone NHEJ or precisely repaired via HDR. **C** The general workflow of plant genome editing

The first breakthroughs involved zinc-finger nucleases (ZFNs) and transcription activator-like effector nucleases (TALENs), which enable a range of genetic modifications (Fig. 1A) (Jaganathan et al. 2018; Mahfouz et al. 2011). ZFNs and TALENs each comprise a sequence-specific DNA-binding module with a specific binding activity, along with a FokI nuclease domain with nonspecific cutting activities (Carroll 2011). The zinc finger proteins, which naturally occur in various organisms, were studied for their ability to bind to specific DNA sequences. Each Cys2His2 zinc finger domain recognizes three base pairs of DNA. Alteration of a small number of residues in or near an alpha-helix within this domain can lead to changes in its DNA-binding specificity (Laity et al. 2001). By arranging multiple zinc fingers together, researchers can create a protein that can recognize and bind to a specific DNA sequence of interest. ZFNs are created by fusing the FokI endonuclease with a zinc-finger protein that can bind DNA (Urnov et al. 2010).

TALENs are similar but structurally distinguishable from zinc-finger proteins because they contain highly variable amino acids at the 12th and 13th positions in their binding domain (Deng et al. 2012; Moscou and Bogdanove 2009). Transcription activator-like effectors (TALEs) are naturally occurring proteins found in certain plant pathogenic bacteria, particularly species of *Xanthomonas* (Romer et al. 2007). These bacteria use TALEs as part of their pathogenicity mechanism to manipulate gene expression within the host plant cells. TALEs have a unique ability to bind to specific DNA sequences (Moore et al. 2014). They achieve this specificity through a modular structure where each TALE protein contains multiple repeating units, generally contains 33–35 highly conserved amino acids. These repeats can differ in just two amino acids, called the repeat variable di-residue (RVD), which determines the binding specificity of each repeat to a single DNA base pair. When a TALE protein binds to DNA, each repeat unit recognizes and binds to one specific base pair in the target DNA sequence. By arranging these repeat units according to the sequence of interest, TALE proteins can bind to any desired DNA sequence with high precision. The TALEs are fused with FokI for targeted nuclease activity. TALENs function in pairs, where two TALEN proteins are designed to bind to opposite strands of the DNA at the desired location, allowing the attached nuclease domains to create a double-strand break (DSB) in the DNA (Cermak et al. 2011; Joung and Sander 2013).

Activation of the FokI nuclease domain requires its dimerization, which is enabled at the target DNA sequence when two modules are created to target closely spaced DNA sequences. This dimerization requirement gives ZFNs and TALENs their specificity; however, designing active nucleases is both expensive and challenging. ZFNs, the first generation of gene-editing technology, were initially employed in animals (Kim and Kim 2014) and later in rice and other crop plants (Ainley et al. 2013; Jung et al. 2018). However, this approach lacks reproducibility and requires complex engineering to select a pair of ZFNs that work in vivo (Kim and Kim 2014). Its use in crops has therefore not been widely developed. TALENs, in contrast, are more precisely targeted, less cytotoxic, do not have a genomic loci effect, and may be assembled in a modular fashion (Briggs et al. 2012). TALENs have been successfully used in crops including wheat (Luo et al. 2019), maize (Liang et al. 2014), and rice (Li et al. 2016). However, the need for guanine nucleotides limits their binding sites, making them unsuitable for repeated editing or use in heavily methylated regions (Cox et al. 2015). Therefore, the use of TALENs is also somewhat constrained.

### CRISPR/Cas9 systems for plant genome editing

CRISPR/Cas (Clustered Regularly Interspaced Short Palindromic Repeats/CRISPR-associated proteins) is an adaptive immune system discovered in bacteria and archaea that helps protect them against invading viruses and plasmids (Barrangou et al. 2007). This system works through a sequence-specific manner to target and cleave the DNA or RNA of invading genetic material. The CRISPR/Cas system consists of two main components; the CRISPR array and Cas proteins (Deveau et al. 2010; van Beljouw et al. 2023; Westra et al. 2014). The CRISPR array is a region of the bacterial genome containing short, repetitive sequences interspaced with unique spacer sequences derived from the genetic material of previous invaders (such as viruses or plasmids) and serves as a memory of past infections. The Cas proteins are CRISPR-associated proteins and are involved in the recognition and cleavage of foreign genetic material. They function by using the information stored in the CRISPR array to identify and target specific sequences in the invading DNA or RNA. The process of CRISPR/Cas immunity involves several steps (Deveau et al. 2010; van Beljouw et al. 2023; Westra et al. 2014). When a bacterium encounters a new viral or plasmid DNA, a portion of it is integrated into the CRISPR array as a new spacer sequence termed as adaptation. Then transcription and processing of the CRISPR array to generate small CRISPR RNAs (crRNAs). Each crRNA contains a spacer sequence and is complementary to the sequence of a specific invading genetic element. Next in the interference, the crRNA guides the Cas proteins to the complementary sequence of the invading DNA or RNA. Finally, the Cas proteins bind to the target sequence and initiate the cleavage or destruction of the foreign DNA or RNA, thus disabling the invader and preventing its replication.

The CRISPR/Cas9 system was developed as a genome-editing tool in 2012 (Mali et al. 2013) and is now the technology most widely used for that purpose. An RNA-guided DNA endonuclease called Cas9 and a single guide RNA (sgRNA) are the two main parts of the CRISPR/Cas9 system (Fig. 1B). The CRISPR-Cas9 system recognizes target DNA sequences through DNA–RNA interaction; the Cas9 protein must be guided to the target site by the sgRNA, which contains 20 nucleotides complementary to the target sequences. In this regard, the method is distinct from ZFN and TALEN genome editing, which recognize target DNA sequences through DNA–protein interaction (Fig. 1A). The CRISPR/Cas9 system requires only the construction of an sgRNA, which can be adapted for diverse genomic targets by the straightforward modification of complementary sequence (Jiang et al. 2013; Shan et al. 2013). Cas9 cleaves the DNA strand after the sgRNA attaches to its complementary protospacer-adjacent motif (PAM) sequence, a brief nucleotide sequence found at the 3′ end of the target sequence near the cutting site. As described before, the CRISPR/Cas9-mediated targeted DSB can be repaired by either error-prone NHEJ or precise-mutation HDR. In the case of HDR, precise mutation can be introduced according to the DNA repair donors with homology arms. However, it’s still a major challenge to induce high-fidelity HDR with a low NHEJ background (Miyaoka et al. 2016).

Since its initial development, the CRISPR-Cas9 system has been further optimized to enhance its specificity and allow precise genome editing with minimal effects on the genome (Jaganathan et al. 2018). Recruitment of Cas9 usually results in DSBs at the target site in the genome; however, unintended changes (off-target effects) are occasionally induced, which is one of the system’s two primary flaws. The other flaw is that its targeting is somewhat restricted due to its requirement for PAMs (Gohil et al. 2021). Thus, improvements to CRISPR/Cas9 that decrease off-target effects and/or provide freedom from PAM restrictions can expand its applications. In particular, various techniques have been shown to significantly decrease off-target effects, including Cas9 modification, sgRNA modification, bioinformatics analysis, and delivery mode optimization (Zuo et al. 2020). Notably, off-target effects have not been observed in plant genomes and are not likely to pose a huge concern.

### The workflow of plant genome editing

The procedure for plant gene editing consists of the following steps (Fig. 1C): (1) a design phase where the sequence of interest for gene editing is selected; (2) the gene editor is chosen and designed accordingly; (3) a build or construction phase where genome-editing vectors are optimized for expression in the target cells; (4) an application phase where the optimized components of the gene-editing system are delivered into the target plant cells; (5) gene editors are expressed and editing the plan genome; (6) genome-edited plants are regenerated; (7) the genotyped plants are phenotyped for the trait of interest in the greenhouse or in field conditions.

The delivery of genome engineering reagents is species-specific and varies among different plant species. Furthermore, regeneration is unique to each plant species and the vector expressing the genome editing reagents must be optimized for expression of the reagents in the target cell as well as the use of a proper selectable marker. Machinery for gene editing may be introduced directly into protoplasts, or directly into plant cells through bombardment with particles containing the appropriate vectors (using a biolistic apparatus, also known as a gene gun) or through infection with an Agrobacterium strain engineered to carry the genes of interest on the T-DNA element. Since the transformation and regeneration methods must be customized for each species and plant variety, which is laborious for most elite varieties and wild species (Altpeter et al. 2016), these processes continue to represent bottlenecks to gene editing in plants.

Agrobacterium-mediated transformation is currently the most effective method to deliver genome editing reagents in DNA form (Liang et al. 2017). When DNA is used, the reagents for genome editing (sgRNA and Cas enzyme) can be expressed transiently or from a transgene that is integrated into the genome as part of a transfer-DNA (T-DNA) construct (Zhang et al. 2016). In the standard method, selective antibiotics are applied during tissue culture to promote the growth of resistant calli expressing the T-DNA genes, and these calli are treated with different hormone regimens that induce them to regenerate into transgenic plants. Transgene-free mutant plants can be obtained by crossing transgenic genome-edited mutants to remove the integrated T-DNA from the mutant genomes.

Alternatively, reagents can be delivered as RNAs (sgRNA and mRNA encoding Cas) or ribonucleoprotein complexes (RNPs, comprising the Cas protein and in vitro–transcribed sgRNA). This avoids any potential integration of foreign DNA into the plant genome and therefore these methods are termed DNA-free (Ran et al. 2017; Zhang et al. 2016) as the mutant plants will lack foreign DNA (Liang et al. 2017; Svitashev et al. 2016). Although, due to the absence of foreign DNA, DNA-free genome editing is preferred to the conventional DNA-based method. However, DNA-free editing generally requires extensive screening to identify editing events due to the absence of selective markers.

## Expanding CRISPR genome-editing platforms for genome manipulation

With the development of CRISPR/Cas systems, a broad range of new Cas effectors and variants has been harnessed for genome engineering in several plant species (Devi et al. 2022; Sedeek et al. 2023; Zheng et al. 2020; Zhou et al. 2020). The identification of new CRISPR/Cas systems has expanded the repertoire of Cas proteins that can target DNA and RNA, making them promising tools for altering plant genomes. Different Cas orthologues with various PAM specificities have been used to enlarge the editing scope of these tools. Various genome editors such as base editors and prime editors are designed for different applications of plant genome editing. In addition, CRISPR/Cas systems have also been explored for genome-scale editing and targeted chromosomal rearrangements. The expanding CRISPR/Cas-based toolkits revolutionized the area of precision genome editing and enabled diverse genome manipulation for crop engineering.

### Discovery of new Cas effectors and orthologues

**Fig. 2:**
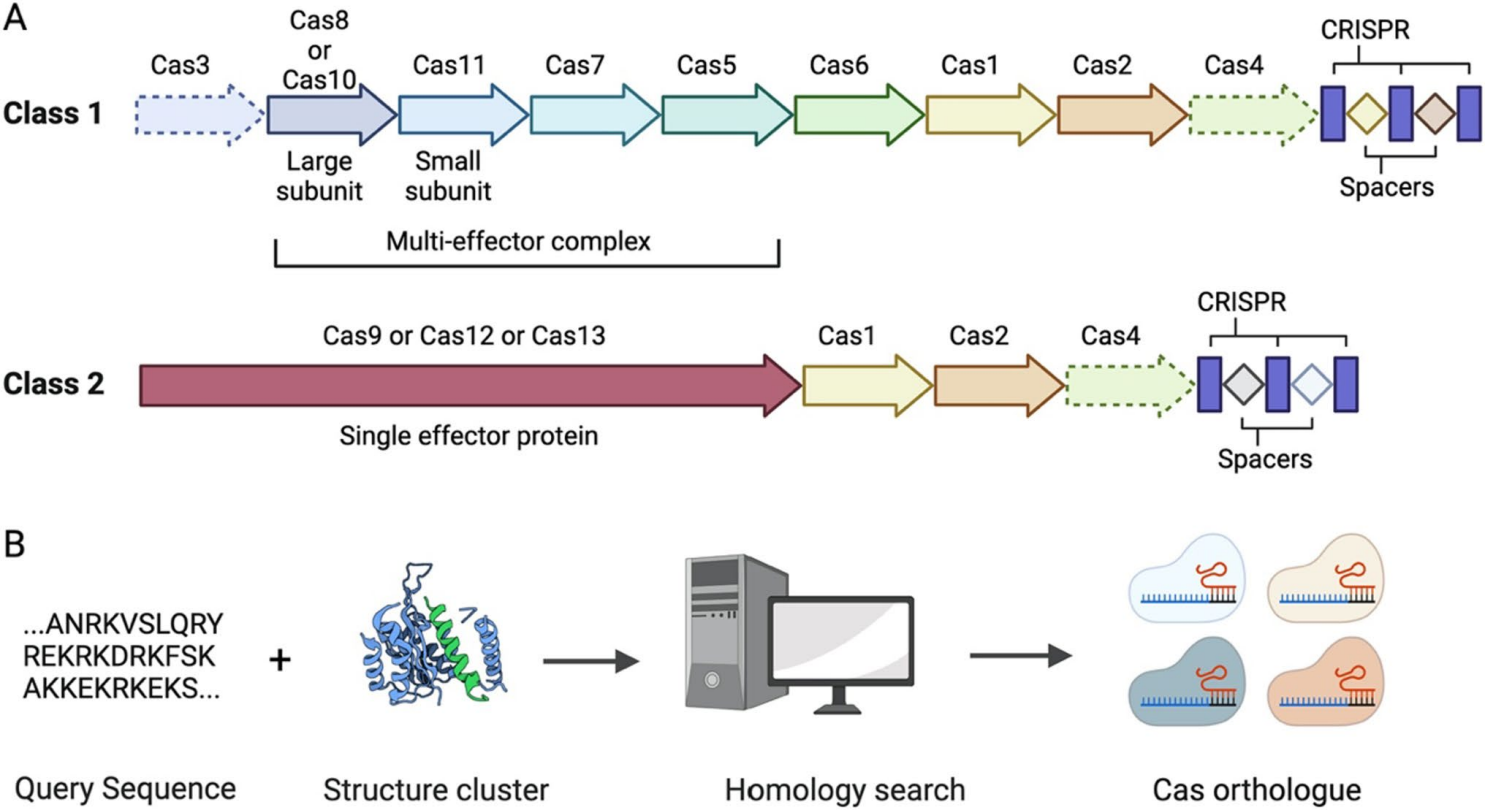
**A** The two classes of CRISPR/Cas system. **B** The sequence for structure-based homology search for new Cas orthologues

To date, CRISPR/Cas systems can be classified into 2 distinct classes, 6 different types, and 33 subtypes (Makarova et al. 2020). In Class I CRISPR/Cas systems, the effector module comprises multi-subunit effector complexes. In contrast, the effector module of Class 2 CRISPR/Cas systems is characterized by singular, extensive multidomain protein effectors such as Cas9, Cas12, Cas13, and Cas14 (Fig. 2A). Besides CRISPR/Cas9 systems, other Class II Cas effectors have also been widely utilized across several domains due to their remarkable attributes, such as heightened sensitivity and exceptional specificity. Among these CRISPR/Cas systems, Cas3, Cas9, Cas12a, Cas12b, Cas12c, and Cas14 targets dsDNA while Cas10, Cas13a, Cas13b, Cas13c, and Cas13d targets ssRNA (Koonin et al. 2017; Qian et al. 2022Xu and Li 2020). The Cas12a and Cas14a also cleave ssDNA (Qian et al. 2022; Wu et al. 2021). The Cas12a and Cas13a also show *Trans* cleavage of ssDNA and ssRNA, respectively (Wu et al. 2021). The identification of new CRISPR–Cas systems has expanded the repertoire of Cas proteins that can target DNA and RNA, making them promising tools for altering plant genomes. In recent years, the CRISPR-CasΦ system, identified in large bacteriophages, has emerged as a new gene editing tool that can specifically target double-stranded DNA and induce staggered cleavage (Pausch et al. 2020). Notably, CasΦ proteins are relatively small, ranging from 700 to 800 amino acids (aa), in comparison to Cas9 (1,000 to 1,400 aa) and Cas12a (1,100 to 1,300 aa). Bacteriophages of considerable size that possess CasΦ systems have been identified in many environments, suggesting that there may be a wide range of optimal temperatures for CasΦ activity. CasΦ proteins are thus intriguing contenders as innovative tools in plant genome engineering (Li et al. 2023).

The effectiveness of Cas effectors is constrained by the specificity of PAM sequences. Many studies have been conducted to discover or engineer new Cas orthologues with better efficiency and differing PAM specificity. Several Cas9 orthologs with varying PAM preferences have been identified in different bacterial species by sequence and structure-based homology search (Fig. 2B). NmCas9 from *Neisseria meningitidis* (Muller et al. 2016), SaCas9 from *Staphylococcus aureus* (Ran et al. 2015), StCas9 from *Streptococcus thermophilus* (Yang et al. 2023a, b), FnCas9 from *Francisella novicida* (Hirano et al. 2016), and CjCas9 from *Campylobacter jejuni* (Kim et al. 2017a, b) are among those discovered so far. The genes responsible for generating most of these proteins are smaller than SpCas9, which is advantageous for gene delivery by viral vectors. Using Cas9 orthologs for simultaneous targeting could facilitate the implementation of multiplex genome engineering, in which multiple genomic regions, each with distinct PAM sequences, are simultaneously targeted. This should enhance the versatility and effectiveness of the CRISPR/Cas system. Meanwhile, the SpCas9 protein is now being engineered to expand its compatibility with PAM sequences or improve its specificity toward PAM sequences while simultaneously minimizing off-target effects (Wada et al. 2020).

### State-of-the-art editors for genome modification

Generating precise genome modifications can be quite challenging, even with CRISPR/Cas systems, due to the low ratio of edits that occur via the HDR repair pathway compared with the NHEJ pathway.

**Fig. 3:**
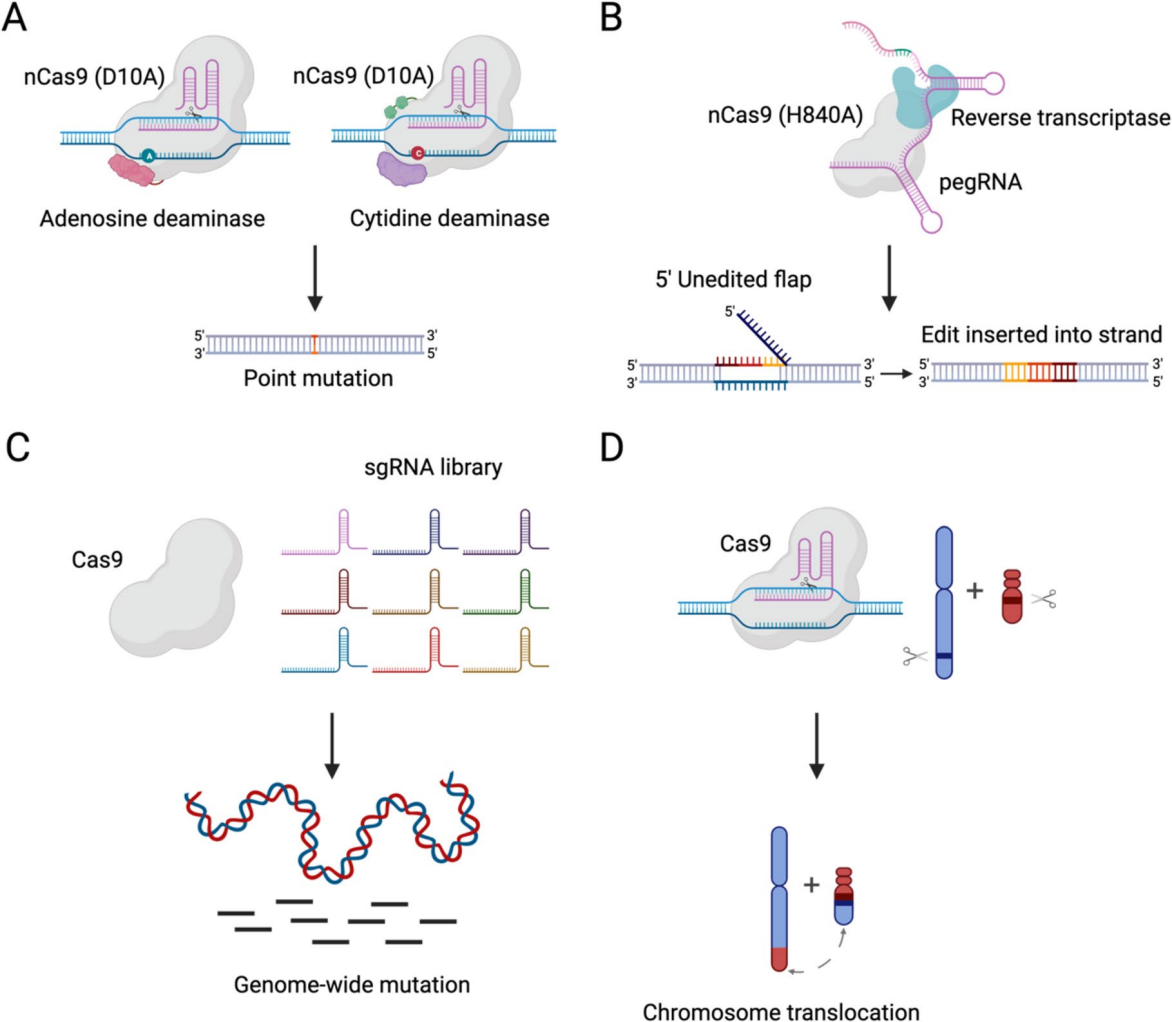
The expanding CRISPR/Cas system-based toolkits. **A** Base editing technology by fusing nCas9 (D10A) with adenosine deaminase or cytidine deaminase for targeted point mutation. **B** Prime editing technology by fusing nCas9 (H840A) with reverse transcriptase and pegRNA for precise genome editing without a double-strand DNA break. **C** Using CRISPR/Cas9 system with sgRNA library for genome-wide mutations. **D** CRISPR/Cas9 system can induce chromosome rearrangement by targeted double-strand breaks

To improve the efficiency of genome editing, various CRISPR-mediated genome editors have been developed. Rather than acting through a DSB, these editors have enzymatic activities that change the properties of nucleotides at the target site. To generate single-nucleotide changes, which are difficult for traditional CRISPR/Cas systems, base editors are a recently developed, highly accurate, genome-editing technology that enables targeted, irreversible conversion of individual bases at desired locations (Fig. 3A) (Gaudelli et al. 2017). Following the introduction of a mutation in Cas9 (D10A), the resulting Cas9 nickase (nCas9) exhibited exclusive capability for single-strand cleavage. Base editor complexes are composed of sgRNA and an nCas9 protein linked to a deaminase domain that can convert specific base pairs (Rees and Liu 2018). Two distinct categories of base editors exist: cytosine base editors (CBEs) and adenine base editors (ABEs). The first class developed was the CBEs, which can convert C to U, thereby converting an uracil-guanine (U-G) base pair into a thymine-adenine (T-A) base pair during the processes of DNA repair and replication. Cytosine deaminase is responsible for modifying bases in CBEs, including uracil glycosylase inhibitors (UGIs) (Gaudelli et al. 2018). In the case of ABEs, Adenine is responsible for replacing the nucleotide base A with I, facilitating the conversion of I-T base pairs to C-G base pairs during the DNA repair and replication processes. Unlike with CBEs, the use of DNA glycosylase inhibitors is not necessary with ABEs. Notably, For both ABEs and CBEs, base editors can only function at a certain distance from the protospacer (editing windows) (Gaudelli et al. 2017). Compared to conventional gene-editing techniques, base editing is more precise with less indel background and is frequently used to modify individual bases within genes.

Base editors are widely utilized in various agricultural contexts, particularly for crop improvement. The most notable and promising application is the development of herbicide tolerance. For example editing specific bases of the gene encoding acetolactate synthase (ALS), a crucial enzyme in amino acid production, leads to tolerance of sulfonylurea and triazolone herbicides in plants (Durner et al. 1991). A Pro-to-Ser amino acid substitution created through CBE editing endowed plants with a tolerance to sulfonamide herbicides, which are used extensively in crop production. The same approach has been used to confer sulfonamide tolerance to crop plants including wheat (TaALS, P174S), watermelon (ClALS, P190S), tomato (SlALS1, P186S), and potato (StALS1, P186S). Notably, transgene-free plants were successfully generated for watermelon, tomato, and potato (Shimatani et al. 2017; Tian et al. 2018; Veillet et al. 2019). The introduction of the mutation AtALS S653N by CBE resulted in tolerance of imidazolinone herbicides. However, this achievement was significantly delayed primarily because the base mutation site was located outside the editing window, causing a notable decrease in editing efficiency. Imidazolinone-tolerant plants have also been successfully developed in Arabidopsis (Dong et al. 2020a, b). Furthermore, herbicide tolerance has been introduced into rice by editing *OsALS* and introducing the C2186R mutation into *Acetyl-coenzyme A carboxylase* (*OsACC*) utilizing the ABEs (Li et al. 2018a, b). Shimatani et al. 2017).

Another technique, prime editing, uses chimeric proteins consisting of a Cas9 nickase domain and an artificially modified reverse transcriptase domain, along with a pegRNA, which provides specificity for targeting and acts as a template for reverse transcriptase to incorporate edits into the genome (Fig. 3B) (Anzalone et al. 2019). After binding to the target site, the Cas9 RuvC nuclease domain initiates a nick in the DNA strand containing PAM. The prime editor subsequently utilizes the recently liberated 3' end at the chosen DNA location to initiate reverse transcription, using the extension present in the pegRNA as a template. For successful priming, the pegRNA extension must possess a primer-binding sequence (PBS) capable of forming a primer-template complex through hybridization with the 3' end of the nicked target DNA strand. Furthermore, pegRNAs are equipped with a reverse transcription template that guides the synthesis of the altered DNA strand at the 3′ end of the target DNA strand. The reverse transcription template comprises the intended alterations in the DNA sequence together with a segment with similarity to the specific location of interest, which aids in DNA repair (Anzalone et al. 2019). Therefore, prime editing enables the introduction of all 12 potential forms of point mutations, encompassing all six feasible base-pair conversions, as well as precise and targeted incorporation of tiny insertions and deletions. This method has a favorable editing-to-indel ratio, making it a promising advance. Thus far, prime editing has been applied to many crops, including rice, wheat, corn, and tomato (Butt et al. 2020; Jiang et al. 2020; Lin et al. 2020; Lu et al. 2021). Nevertheless, the technique’s efficiency in plants is considerably lower than that observed in human cells. Consequently, several approaches have been implemented to enhance its efficiency. Jiang et al. (Jiang et al. 2020) and Lin et al. (Lin et al. 2020) tested several modifications to the elements of prime editing machinery, such as optimizing the length of the pegRNA, modifying the designed reverse transcriptase, and improving the pegRNA promoter. Lin et al. (Lin et al. 2021) also employed distinct pegRNA sequences in the template and antisense strand. These approaches substantially enhanced the efficiency of prime editing in plants. Anzalone et al. (Anzalone et al. 2022) showed that pairs of pegRNAs could accurately remove a segment of 710 bp or replace a sequence of 108 bp. The full potential of prime editing has yet to be thoroughly realized.

### CRISPR-Cas-mediated genome-scale engineering

Traditional methods for random mutagenesis have provided substantial insight into gene function. However, genetic redundancy has prevented researchers from addressing gene function for complex, multi-gene families. Now, genome-scale engineering allows the simultaneous generation of many targeted mutations genome-wide. For example, Multi-Knock uses the CRISPR toolbox to address functional redundancy in Arabidopsis by targeting multiple gene family members simultaneously (Fig. 3C) (Hu et al. 2023; Zhang et al. 2021). Multi-Knock enables the identification of genetically concealed components within the plant’s genome. A total of 59,129 sgRNAs were computationally generated, with the objective of simultaneously targeting 2 to 10 genes within a given family (Hu et al. 2023). Among them, 5,635 sgRNAs were used to target the plant transportome. This approach generated more than 3,500 distinct Arabidopsis lines, which proved crucial in the identification and characterization of the first cytokinin tonoplast-localized transporters. The approach enables scientists and breeders to effectively address functional redundancy in plants at the genome level to accelerate breeding efforts.

In a more recent study, He et al. successfully showcased the viability of employing a pooled CRISPR library for genome-scale targeted editing in *Brassica napus*, an allotetraploid crop (He et al. 2023). A comprehensive set of 18,414 sgRNAs was developed with the purpose of targeting 10,480 specific genes of interest. Subsequently, a total of 1,104 regenerated transgenic plants were successfully grown, which included 1,088 distinct sgRNAs. Analysis of the resulting data indicated that out of 178 genes examined, 93 carried mutations. This finding suggests an editing effectiveness of 52.2%. Notably, it has been observed that Cas9-mediated DNA cleavages tend to occur at all target sites directed by a single sgRNA in polyploid plants. The authors also demonstrated the robustness of reverse genetics screening in identifying diverse features using post-genotyped plants, discovering several genes that influence the plant’s fatty acid profile and seed oil content, effects that had not been previously documented. These findings offer significant contributions to the fields of functional genomics and elite crop breeding and demonstrate that the pooled CRISPR library approach is an excellent tool for genome-scale targeted mutagenesis in plants.

### CRISPR-Cas-mediated chromosome engineering

The presence of genetic linkage makes it difficult to transfer desirable traits from wild species to a crop cultivar. Meiotic recombination can address this issue by crossovers between parental homologous chromosomes (Lambing et al. 2017). However, due to the inherent limitations of, and lack of control over, the rate and distribution of natural crossovers, significant portions of the chromosomes are excluded from participating in genetic exchange. Therefore, the targeted chromosome rearrangement induced by CRISPR/Cas systems have the potential to boost the meiotic rearrangement for crop breeding (Ronspies et al. 2021).

The adaptability of genome-editing tools to manipulate meiotic recombination was initially demonstrated by recruiting the CRISPR/Cas system with SPO11 (a natural inducer of meiotic DSBs) in yeast (Sarno et al. 2017). By directing the SPO11 fusions toward regions with inherently low levels of DSB induction during meiosis, a noticeable occurrence of SPO11-mediated DSB creation and a substantial rise in crossover frequency were observed. In recent studies, megabase pair (Mbp) chromosome rearrangement has also been achieved in *Arabidopsis thaliana* (Schmidt et al. 2020). By the egg-cell-specific expression of the Cas9 nuclease, a targeted reversal of the 1.1 Mb long hk4S-inversion can be achieved. meiotic crossovers can be restored because of genetic linkage breakage. Following this strategy, CRISPR/Cas system-mediated Mbp inversion was recently achieved in crops. Chris et al. applied CRISPR/Cas9 technology for targeted 75.5-Mb pericentric inversion in maize (Schwartz et al. 2020). These studies unlock a large chromosomal region for recombination. Reciprocal translocations in plants were recently achieved by CRISPR-Cas9 mediated induction of DSBs (Beying et al. 2020). Beying et al. induced heritable 1 MPb level translocations with up to 2.5% in the wild-type *Arabidopsis thaliana*, and up to 3.75% in NHEJ mutant ku70 *Arabidopsis thaliana* (Fig. 3D). The development of chromosome engineering tools would allow us to release the potential of chromosomal rearrangements for plant breeding, reconstruct chromosome sets of the ancestors of current plant species, and even create new plant species.

## Harnessing gene editing for crop breeding

**Fig. 4:**
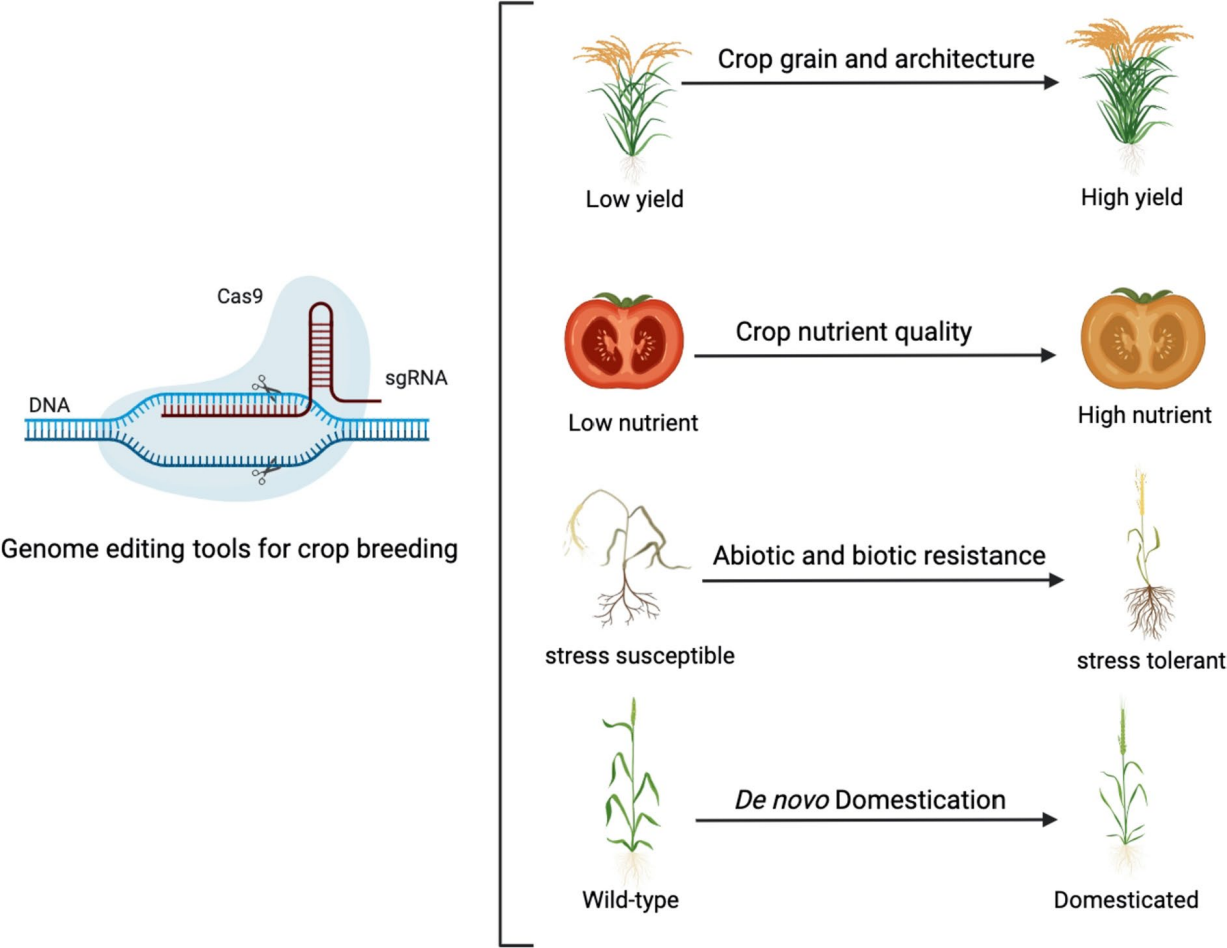
The promising application of gene editing tools for crop breeding

Harnessing valuable genetic divergence and eliminating maladapted genetic elements are central challenges in crop breeding (Rao et al. 2021). In plant biotechnology, researchers have successfully introduced specific genes of interest into various crop lines through different kinds of modifications such as loss of function, gain of function, altered expression, and protein truncation. These genetic manipulations have successfully led to novel crop varieties that exhibit desirable traits across diverse plant species (Zhang et al. 2018a, 2018b). Gene-editing technology presents a promising avenue for efficiently transforming unfavorable variations into favorable ones (Fig. 4). In this section, we aim to illuminate the diverse applications of gene editing in crop breeding, specifically focusing on the enhancement of essential agronomic traits including heightened productivity and nutrients, increased abiotic and biotic resistance, and the novel pursuit of de novo domestication.

### Improving crop productivity via gene editing

To resolve global food security, researchers have dedicated efforts to enhance crop productivity and nutrients. Nevertheless, traits influencing crop productivity are intricate and often involve the interplay of multiple genes. These traits encompass various factors like grain yield and plant architecture.

The intricate regulation of grain yield is orchestrated by quantitative trait loci (QTLs), which typically exert influence over several characteristics, including thousand-grain weight (encompassing grain size), the count of grains per panicle, the number of florets per panicle, and the number of panicles per plant. To date, 19 QTLs modulating grain size in rice have been successfully cloned and described. For instance, CRISPR-based knockdown of *OsGS3* and *OsGL3.1* in rice resulted in the enhancement of grain size and, thus, the thousand-grain weight and the overall yield per plant (Usman et al. 2021). Applying multiplex gene-editing techniques targeting three specific genes (*OsGS3, OsGW2*, and *OsGn1a*) enhanced grain production in edited rice lines compared to their wild-type counterparts. Wang et al. successfully induced a significant increase in seed size and grain weight by introducing heritable mutations into the *TaGW2*, *TaLpx-1*, and *TaMLO* genes within hexaploid wheat (Wang et al. 2018). The architecture of the crop panicle plays a crucial role in determining the number of grains per panicle and, consequently, influences overall grain yield. Numerous genes responsible for the regulation of panicle have been identified over the past few decades (Li et al. 2021). By mutating DENSE AND ERECT PANICLE1 (DEP1) by the CRISPR/Cas9 system, Huang et al. created mutant alleles that resulted in yields surpassing those conferred by other naturally occurring high-yield alleles in rice (Huang et al. 2009).

Plant architecture encompasses the structural arrangement of plant organs such as stem height, branching pattern, leaf shape, inflorescence distribution, and root structure which affects crop yields through both genetic and environmental factors (Huang et al. 2021). Rice plants with reduced height have also been successfully produced (Wan, et al. 2022). In another study, deploying CRISPR/Cas9 technology to specifically disrupt the gene *CAROTENOID CLEAVAGE DIOXYGENASE 7* (*CCD7*) resulted in a noteworthy enhancement of tillering, accompanied by a decrease in plant height (Butt et al. 2018; Ren et al. 2020). Plant height is also crucial due to their allocation for biomass and node spacing (leaves, branches, reproductive organs). CRISPR/Cas9 was employed to generate loss-of-function alleles of GA20ox2 in rice, replicating the sd1 mutation's effects, resulting in reduced stature (22.2%) and increased yield (6%) without impacting other agronomic traits (Han et al. 2019). A double rice mutant was created by simultaneously editing the MIR396 gene family, *MIR396e* and *MIR396f,* two important regulators of grain size and plant architecture. This mutant exhibited enhanced grain yield and flag leaf area, which was attributable to an elevated concentration of mevalonic acid, a precursor to the plant hormone gibberellin (Miao et al. 2020). Additionally, CRISPR-based editing of the gene *OsLOGL5*, encoding a cytokinin-activation enzyme, resulted in a suppressive effect on root growth and tiller number and overall growth enhancement (Wang et al. 2020). Besides, the phenomenon of pod cracking in grain crops presents substantial risks to crop productivity. In a recent study, the gene *qSH1* was subjected to CRISPR-Cas9-mediated mutagenesis to develop rice lines with enhanced resistance to shattering (Sheng et al. 2020).

### Enhancing nutrient quality via gene editing

CRISPR/Cas genome editing technique has been extensively explored for the nutritional enhancement of crops. As the major corn storage proteins, Zeins, are deficient in lysine and tryptophan (essential amino acids), contribute to insufficient nutritional quality. To overcome this issue, CRISPR/Cas9 system was employed to target the 19 kDa alpha zein gene family, facilitating proteome rebalancing without a complete knockout of alpha-zein, leading to an increase of up to 30% in the lysine content (Hurst et al. 2023). Gluten, the storage protein of wheat, can cause celiac and non-celiac disease and gluten ataxia. Through α-gliadin genes knockout by CRISPR/Cas9 system, mutated wheat was found to have low gluten content and reduced immunoreactivity (Sánchez-León et al. 2018). In oilseed crops, the CRISPR/Cas12a (formerly known as Cpf1) has been employed in soybeans to manipulate the *FAD2*-1B and *FAD2*-1A genes to improve oil composition by developing soybean plants bearing seeds with increased oleic acid levels, enhancing oil yield (Kim et al. 2017a, 2017b).

In addition to the knockout strategy, knock-in approaches have also been utilized for nutrient enhancement. For instance, the Golden rice cultivar Kitaake has been developed by Knock-in, a 5.2-kb carotenogenesis cassette consisting of *CrtI* and maize *PSY* genes. The variety contains 7.9 μg/g dry weight (DW) β-carotene in the endosperm (Dong et al. 2020a, 2020b). Introducing the cauliflower mosaic virus 35S promoter upstream of the anthocyanin mutant 1 gene (ANT1, a Myb transcription factor) in the tomato genome through knockin techniques yielded purple tomatoes characterized by elevated anthocyanin content (Ku and Ha 2020).

### Improving abiotic resistance via gene editing

CRISPR technology has been extensively employed to convey tolerance of various stresses to numerous crops, including wheat, rice, maize cotton, soybean, tomato, and potato. CRISPR has revolutionized plant breeding efforts, enabling the development of adaptive climate-tolerant crops. CRISPR/Cas9 was used to target the eukaryotic translational initiation factor *eIF4E* gene, an essential component in the translation mechanism, in winter barley, melon, and hexaploid wheat (Hoffie et al. 2021; Kan et al. 2023; Pechar et al. 2022). The resulting deletion conveys resistance to Bymovirus Resistance in Winter Barley, Moroccan watermelon mosaic virus in melon, and yellow mosaic virus in wheat (Hoffie et al. 2021; Kan et al. 2023; Pechar et al. 2022).

Elevated salt concentrations in the soil substantially undercut crop production. The mechanism behind salt tolerance was elucidated using CRISPR/Cas9 technology through disruption of the target genes. ABA-induced transcription repressors (AITRs) are a novel family of transcription factors and key regulators of ABA signaling. In soybean, the *gmaitr* double and quintuple mutants showed tolerance to salt stress at germination and seedling stages (Wang et al. 2021). PARAQUAT TOLERANCE 3 (PQT3) is an E3 ubiquitin ligase and loss-of-function mutants generated via CRISPR/Cas9 system showed enhanced tolerance to oxidative and salt stress under control as well as field conditions (Alfatih et al. 2020).

CRISPR-Cas9 advanced breeding facilitated the development of a novel variation of *Auxin-Regulated Gene Involved in Organ Size 8* (*ARGOS8*) in maize that enhances grain yield under drought conditions compared to wild-type. This study supports the status of the CRISPR/Cas9 system as a precise and effective method for creating novel allelic variants in crops, with the specific aim of enhancing drought tolerance in plants (Shi et al. 2017).

In addition, the CRISPR/Cas9 technology was utilized to disrupt the expression of *Robust Root System 1* (*RRS1*) in rice. The loss-of-function mutant of *RRS1* enhances drought tolerance by improving water use efficiency and promoting water absorption (Gao et al. 2023). Molecules such as mitogen-activated protein kinases play crucial roles in tomato by protecting membrane cells from oxidation and regulating transcription genes to mitigate the effects of drought stress. Wang et al. (Wang et al. 2017) documented the regulation of drought tolerance through the gene *Mitogen-Activated Protein Kinase 3* (*SlMAPK3*) in tomato, employing CRISPR/Cas9 technology to generate knockout mutants of *SlMAPK3* to investigate its response to drought stress. Global warming generates heat stress that affects plant growth and development and ultimately affects crop yield. CRISPR/Cas9-mediated gene editing of *Agamous-Like 6* (*SlAGL6*, belongs to MADS‐box gene family) decreased the sensitivity of tomatoes to heat stress (Klap et al. 2017). Similarly, *Thermo-tolerance 3* (*TT3*) comprises two genes, *TT3.1* and *TT3.2*, that regulate thermotolerance in rice. The *tt3.2* mutant allele generated through the CRISPR/Cas9 system is tolerant to heat stress (Zhang et al. 2022).

### Improving biotic resistance via gene editing

Biotic stress in crops, such as pathogens and weeds, refers to the adverse impact of living organisms on plant health and productivity. In recent years, the utilization of CRISPR-Cas technology has been associated with substantial improvements in plant traits by modifying gene regulation to enhance various biotic resistance traits (Ghosh and Dey 2022; Maharajan et al. 2022).

Pathogens, such as viruses, bacteria, and fungi, are obligate parasites with a significant capacity to infect plants, resulting in a substantial decline in crop productivity ratios. Through CRISPR/Cas systems, pathogen resistance can be achieved by knocking out the pathogenicity-related genes. By targeting *nCBP-1* gene and *nCBP-2* gene which helps viral replication in *Manihot esculenta*, Gomez et al. successfully generated *Cassava brown streak virus* (CBSV) resistant lines (Gomez et al 2019). Similarly, Chandrasekaran et al. developed multiple viral-resistant cucumbers by knocking out the *eIF4E* gene that helps viral replication (Chandrasekaran et al. 2016). Oliva et al edited sucrose transporter genes (*SWEET11*, *SWEET13* and *SWEET14*) of rice, leading a broad-spectrum resistance to *Xanthomonas oryzae pv. Oryzae (*Oliva et al. 2019*).* The mildew resistance locus O (MLO) is a recognized component of the plant defense system acting as a host S gene. The mutated homo-alleles of MLO in wheat plants—specifically, *TaMLO-A1*, *TaMLO-B1*, and *TaMLO-D1*, exhibited resistance to *Blumeria graminis f. sp. tritici*, the causal agent of powdery mildew disease (Wang et al. 2014). Other than editing host-susceptible genes, the CRISPR/Cas system can be harnessed to directly target pathogen sequences for resistance. By inactivating the targeted virus sequence, transgenic banana lines exhibited achieved resistance to endogenous banana streak virus (eBSV) (Tyagi et al. 2021).

Weeds represent another significant biotic stress for plants due to the substantial damage, particularly the competition for space, sunlight, water, and fertilizers. By editing the *LsGGP2* gene, paraquat-resistant lettuce with high ascorbic acid was developed against oxidative stress as an herbicide-resistant crop (Zhang et al. 2018a, 2018b). Similarly, herbicide resistance can be achieved by point mutations in the 548th and 627th amino acid positions of the acetolactate synthetase (ALS) gene in rice (Sun et al. 2016). The mutagenesis of the tomato plant's CCD8 gene using CRISPR/Cas9 resulted in the creation of a crop resistant to the Phelipanche aegyptiaca weed (Bari et al. 2019).

### De novo domestication via gene editing

In sustainable agriculture, introducing new crops, such as formerly marginal or ‘orphan’ species that are well-adapted to marginal lands and serve as sources of nutrition can complement strategies to improve current crops. All of today’s key crops are descended from wild ancestors and have undergone long domestication processes of enrichment for productivity-improving traits, including optimal plant architecture, high yield capacity, and easy harvestability. However, this long evolutionary process eventually leads to genetic bottlenecks, which narrow the genetic diversity of species and diminish the plants’ tolerance to different abiotic and biotic stresses.

More recently, the strategy of crossing desirable traits from wild relatives into domesticated crops to enhance them has been adopted. However, crossing and backcrossing can only be used to fix monogenic traits, whereas many desirable characteristics in wild species, such as tolerance of abiotic stresses, are polygenic and thus difficult to fix through segregation. A proposed alternative breeding technique, the neo-domestication of wild species through genome editing, might circumvent this difficulty (Chen et al. 2021). Also known as de novo domestication, neo-domestication is the conversion of crop wild relatives, or any other potentially economically important wild plants, into sustainable commodity plants. As a proof of concept, the wild tomato species known as currant tomato (*Solanum pimpinellifolium*) was partially domesticated through multiplex editing of crucial domestication genes while retaining the stress resilience of the wild accessions (Li et al. 2018a, 2018b; Zsogon et al. 2018).

The domestication of wild ancestors of major cereal crops such as rice is another interesting target. The wild rice *Oryza alta*, whose useful traits include high biomass and resistance/tolerance to different biotic and abiotic stressors, was de novo domesticated as a potential future crop species. CRISPR gene editing was then used to select and produce mutant lines of *O. alta* with desired commercial characteristics. Research to date has paved the way for the de novo domestication of many other wild plant species (Yu et al. 2021).

De novo domestication also promises to boost orphan crops’ productivity and nutrient levels to meet specific regional demands. Orphan crops are wild plants that produce substantially lower yields than domesticated varieties but better tolerate environmental challenges and, in many cases, can be planted on marginal terrain (Chen et al. 2021). Accelerated domestication was successfully demonstrated for the orphan crop ground cherry (*Physalis pruinosa*), a close relative of tomato (*Solanum lycopersicum*) (Lemmon et al. 2018). Other orphan crops that may be suitable starting materials for neo-domestication include various “heirloom” Poaceae species, such as millet (*Setaria viridis*), fonio (*Digitaria* spp.), finger millet (*Eleusine coracana* L. Gartn.), triticale (x *Triticosecale Wittmack*), spelt (*Triticum spelta* L.), rye (*Secale cereale* L.), teff (*Eragrostis tef*), quinoa (*Chenopodium quinoa*), cassava (*Manihot esculenta* Crantz), and cowpea (*Vigna unguiculata*). Iterative editing, conventional breeding, and other techniques will be needed to establish completely domesticated versions of orphan crops (Van Tassel et al. 2020).

## Challenges and prospects for the future

### Enhancing the efficiency and precision of genome-editing technologies

Despite recent advances in plant genome engineering technologies, it remains challenging to achieve all necessary modifications inside a genome. More precise genome-editing techniques are needed to facilitate crop improvement, specifically through targeted base substitutions, gene insertions/deletions, and gene substitutions. HDR-mediated genome editing can potentially modify any genome and accurately generate a desired alteration. Several approaches have been used to enhance the efficiency of HDR in plant cells. These include the utilization of geminivirus constructs (Baltes et al. 2014), the implementation of a plant gene-targeting system (Fauser et al. 2012), the use of RNA-templates for DNA repair (Butt et al. 2017), the use of chemical modifications to stabilize donor templates (Lu et al. 2020), the positioning of the donor DNA template in close proximity to the DSBs (Ali et al. 2020), the manipulation of DNA repair pathways (Christian et al 2013), the exploitation of specific cell cycle phases and cell types (Wolter et al. 2018), and the use of different single-strand nucleases (Merker et al. 2020). Indeed, the fact that so many varied approaches have been tested points to the fact that the efficiency of HDR-mediated genome editing in somatic plant cells remains notably low (Steinert et al. 2016).

To address these constraints, the recently devised DNA base-editing systems offer effective, straightforward approaches to convert a certain DNA base into another base at a specific genomic site. Importantly, however, these methods are presently restricted to substitutions involving C-G to T-A and A-T to G-C (Chen et al. 2019). Hence, it is imperative to develop alternative base-editing methodologies, such as the C-G to G-C conversion (Kurt et al. 2021), by engineering deaminases, modifying the DNA repair pathway, and/or employing protein engineering techniques. In addition, prime editing has emerged as a potential alternative method for generating base substitutes. However, existing implementations of prime editing demonstrate rather poor editing efficiency. Given the multitude of parameters influencing the efficacy of a prime editor, including the activity of the reverse transcriptase, the length of the PBS in the pegRNA, and the template for reverse transcription, additional techniques are needed to enhance prime editor activity in plant cells. While prime editing is limited in its ability to generate large gene insertions, a recent breakthrough in the form of CRISPR-associated transposases has demonstrated high efficiency in integrating DNA into bacterial genomes (Klompe et al. 2019; Strecker et al. 2019). This discovery holds promise for the potential future application of large DNA insertion into plant genomes.

### Enhancing the specificity of genome-editing technologies

Off-target consequences are a primary concern in genome editing. CRISPR technologies give rise to two distinct categories of off-target edits: sgRNA-dependent and sgRNA-independent off-target edits. Off-target events dependent on sgRNAs are caused by the editing of off-target locations that have nucleotide mismatches with the on-target sgRNA sequence. Several CBEs have been found to cause off-target mutations throughout the genome in rice, independent of sgRNA (Jin et al. 2019). These changes are initiated by the cytidine deaminase activity in ssDNA regions across the genome. In contrast to standard mutation breeding methods that introduce numerous unwanted mutations into the plant genome, plant genome-editing techniques exhibit high specificity. Furthermore, backcrossing can effectively mitigate the occurrence of limited off-target mutation events. CRISPR–Cas specificity can be enhanced through various methods, as exemplified in studies conducted on wheat and maize (Liang et al. 2017; Svitashev et al. 2016). These include the transient expression of editing reagents, the use of guide RNAs that are deliberately designed (Zhang et al. 2019), and the use of engineered, precise variants of Cas9, Cas12a, and deaminases (Jin et al. 2020). Nevertheless, additional investigation is required to tackle the tendency for unintended modifications to occur and to refine techniques for discovering off-target genetic changes throughout the plant genomes. Furthermore, it is crucial to identify enhanced or novel editing tools with greater specificity. As the technology progresses, concerns regarding the off-target impacts of plant genome editing should be mitigated.

### Delivering genome editing reagents into plants

The current toolbox of CRISPR reagents enables the manipulation of genes through mechanisms including gene knockouts, gene insertion, base editing, and multiplexing. Even so, the biggest challenge in plant genome editing is the successful delivery of the reagents into the plant cells. Plants have a larger, more complex genome architecture than other organisms, which is mostly attributed to the common incidence of polyploidy along with additional genome rearrangements. Moreover, the hard cell walls surrounding plant cells are obstacles to the penetration of external macromolecules. Genetic transformation for the delivery of gene-editing reagents has been successfully achieved in only a restricted range of plant species.

There are three primary techniques employed for the genetic transformation of plants: agrobacterium-mediated transformation, biolistic transformation, and PEG-mediated transformation. Agrobacterium, however, infects only a limited range of hosts, and some species are resistant to Agrobacterium-mediated transformation. Particle bombardment has demonstrated superior efficacy over Agrobacterium transformation in co-delivering cargo for simultaneous editing (Kuang et al. 2020). Nevertheless, the complicated segregation patterns of DNAs integrated into plant genomes through bombardment may pose challenges to the subsequent utilization of genetically edited plants. Using Agrobacterium and biolistic transformation techniques for manipulation of pollen overcomes the need for regeneration; however, it can reduce pollen viability (Liang et al. 2017). Although advances in pollen magnetofection help address these limitations, this technique remains limited to dicots (Vejlupkova et al. 2020). Nanoparticles are another promising vehicle for delivering plant genome-editing tools (Demirer et al. 2021), but additional technical advances are necessary to effectively support nanoparticle-based gene editing in plants.

Except in Arabidopsis and closely related species that can undergo floral dip transformation, the transformation of most crops must be followed by tissue culture to regenerate viable plants from somatic cells. This poses a significant impediment in numerous agricultural crops because the process is laborious and most of the species are recalcitrant to callus induction and regeneration. The promotion of cellular proliferation often depends on the use of a growth medium supplemented with hormones. The hormones induce cell division and maintain the resulting callus at a comparable cell concentration to meristematic cells. However, the concentrations needed differ among species and must be individually tuned. One significant advance has been the enhancement of tissue culture efficiency through the overexpression of growth factors: IPT in dicots (Qin et al. 2011) and Wus2 in monocots (Lowe et al. 2016). Overexpressing two developmental regulators, WUSCHEL (WUS) and BABY BOOM (BBM), improves regeneration frequencies in various transformation-recalcitrant genotypes and species (Lowe et al. 2016). GROWTH-REGULATING FACTORs (GRFs), GRF-INTERACTING FACTORs (GIFs), and GRF-GIF chimeras have also been used to improve the regeneration efficiency of various monocot and dicot plants (Debernardi et al. 2020). In contrast to WUS and BBM, GRFs, GIFs, and GRF-GIF chimeras have no apparent side effects when they are constitutively expressed. The GRF4-GIF1 are fused with CRISPR/Cas9 to produce edited plants with higher regeneration efficiency (Debernardi et al. 2020). Recently a GGB transformation system was established with sevenfold higher transformation efficiency in maize (Chen et al. 2022). In the GGB transformation system, the BABY BOOM transcriptional regulator (ZmBBM/EREB53) and the wheat GRF4-GIF1 (GROWTH REGULATING FACTOR4-GRF-INTERACTING FACTOR1) are combined to boost the regeneration efficiency without any side effects on plant development. The developments for efficient transformation and regeneration will promote the delivery of gene editing reagents in plants.

### Tissue culture-free gene editing in plants

The primary obstacle to fully harnessing the potential of gene editing in plants is the dependence on tissue culture–based genetic transformations. Recent progress with delivery mechanisms, such as de novo meristem induction and the use of viral vectors to bypass tissue culture, is dependent on the use of Agrobacterium for delivery. However, these developments have been demonstrated solely in dicots and still need to be extended to monocots.

A more recent strategy to circumvent the need for tissue culture involves the application of an RNA virus vector derived from *Nicotiana tabacum rattle virus* (Ellison et al. 2021). The virus was genetically modified to generate an sgRNA that was linked to the mRNA of *FT*, which encodes a flowering factor that can undergo intercellular and long-distance movement through the phloem and even traverse grafting junctions across different species. *Nicotiana tabacum* harboring the *Cas9* gene were employed as transgenic lines for the purpose of virus infection. The researchers successfully achieved gene editing in the infected leaves and observed elevated editing rates in the upper regions of the plants compared to the initial infection site. In principle, the application of RNA virus-mediated distribution of the sgRNA might also be extended to include base-editing and prime-editing techniques.

Ma et al. (Ma et al. 2020) proposed an alternative approach to achieve DNA-free editing of somatic plant cells by utilizing sonchus yellow net rhabdovirus (SYNV), which has a large cargo capacity as a vector for the expression of the Cas9 nuclease and sgRNA. The utilization of RNA viruses in crop applications requires them to accommodate many kilobases of surplus genetic material. However, this rhabdovirus has a limited host range. Regrettably, the majority of RNA viruses lack the capacity to accommodate enough additional genetic material for encoding a protein as large as Cas9. However, a recent study showed that smaller CRISPR/Cas nucleases, such as CasΦ, can be used for editing purposes (Pausch et al. 2020). In addition, the use of smaller Cas9 effectors could effectively mitigate the limited cargo capacity of certain viral vectors. This suggests that RNA viruses in crop applications may hold significant potential for future applications.

A tissue culture-independent strategy was applied to produce gene-edited plants via induction of de novo meristems (Maher et al. 2020). According to Maher et al. (2020), it is possible to regenerate genome-altered shoots from soil-grown tobacco without the need for a tissue-culture stage. The utilization of this methodology possesses the capacity to reduce expenses and time associated with the production of genetically modified crops. A recent study used *Agrobacterium rhizogene* to transform the explants that generate transformed roots. The delivery system termed cut–dip–budding (CDB) produces transformed buds due to root suckering under non-sterile conditions and without the need for tissue culture (Cao et al. 2023). The CDM is used to establish gene editing in several species including woody, herbaceous, and tuberous root plants.

In another approach, transgene-free plants were produced in a single step via grafting the wild-type shoots (scions) to the transgenic rootstocks that harbors CRISPR/Cas9 cassettes (Yang et al. 2023a, 2023b). The Cas9 and guide RNA transcripts fused to tRNA-like sequence motifs that move RNAs from transgenic rootstocks to wild-type shoots and achieve heritable gene editing. This method shortens the time to produce transgene-free plants in breeding programs, however, one cannot escape the generation of transgenic rootstocks. It is anticipated that the coming years will bring an increasing array of alternatives for plant genome editing that do not involve the use of DNA.

### Regulation of gene-edited crops

A global debate has arisen over the management of genetically modified crops created using gene-editing technologies. Currently, the regulation of gene-edited crops varies substantially between jurisdictions. A few nations have established guidelines or legislation concerning gene-edited crops. The U.S. Department of Agriculture (USDA) issued a ruling in 2018 stating that genetically edited crops are exempt from further examination by the USDA as long as they do not involve plant pests or contain foreign DNA from plant pests, such as viruses or bacteria (APHIS 2018; Turnbull et al. 2021; Wolt and Wolf 2018). Nevertheless, the regulatory control of gene-edited crops may be linked to their specific genetic properties, requiring the approval of regulatory bodies such as the Environmental Protection Agency (EPA) and/or the Food and Drug Administration (FDA). Three years ago, the SECURE (sustainable, ecological, consistent, uniform, responsible, efficient) platform was launched to enhance and modernize the biotechnology approval process in the United States (Barrangou 2020).

In contrast to the United States, the European Union has determined that the regulatory framework regulating gene-edited crop release should be aligned with the existing laws applicable to genetically modified organism (GMO) crops and products. Other countries have adopted more complicated approaches. In Australia, gene-edited crops are classified into three categories: SDN-1, for gene editing involving point mutations; SDN-2, characterized by the incorporation or editing of a few base pairs using an external DNA template sequence; and SDN-3, for gene editing in which a longer DNA fragment or gene is inserted. Each category is subject to distinct restrictions. For instance, type SDN-1 edits are excluded from regulation by the Office of the Gene Technology Regulator (OGTR), whereas type SDN-2 and SDN-3 edits are subject to regulation (Menz et al. 2020). Canada employs a regulatory framework in which the assessment of a product is based on the end result (outcome) of the genetic change rather than the editing technique used to produce it. Therefore, crops that have undergone gene editing are subject to regulation under the category of plants with novel traits (PNTs). Irrespective of the method employed for their creation (e.g., conventional breeding, transgenesis, mutagenesis, or gene editing), newly developed crops with unique traits must undergo environmental and safety evaluations per the existing standards (Smyth 2017). China's policy regarding genome-editing research is distinct due to the notable absence of regulatory measures on gene-modified crops, despite substantial investments in this field (Lyzenga et al. 2021). The current state of gene-editing regulation worldwide is characterized by a lack of stability and a pressing need for revision in numerous countries. The global perception of the intricate aspects of gene editing will significantly influence this technology's implementation and international trade.

## Conclusions

The emergence of genome-editing techniques in plant science has opened up a wide range of possibilities for advancing plant breeding methods. The use of genome-editing techniques for mutagenesis has established a solid foundation for many advanced breeding approaches, which hold the potential to transform the agricultural landscape significantly in the near future. To fully harness the capabilities of plant genome editing, it is imperative to thoroughly investigate all available methodologies. Genome editing enables the deliberate integration of desired genetic characteristics into crops through a rational approach. Using these accurate and efficient procedures in the context of speed breeding yields outcomes comparable to those achieved with conventional breeding methods. Nevertheless, the replacement of traditional methods by genome-editing-based next-generation breeding is improbable. The widespread adoption of genome editing in agriculture can only be ensured when it is integrated with other technologies, including high-throughput phenotyping, genomic selection, and speed breeding. Adopting a multidisciplinary approach can enhance the field of plant breeding, thereby helping make a second Green Revolution a reality. This will be crucial to effectively address the escalating food requirements of a rapidly expanding global population, particularly in the face of continuously shifting climate patterns.
